# P21 Deficiency Delays Regeneration of Skeletal Muscular Tissue

**DOI:** 10.1371/journal.pone.0125765

**Published:** 2015-05-05

**Authors:** Nobuaki Chinzei, Shinya Hayashi, Takeshi Ueha, Takaaki Fujishiro, Noriyuki Kanzaki, Shingo Hashimoto, Shuhei Sakata, Shinsuke Kihara, Masahiko Haneda, Yoshitada Sakai, Ryosuke Kuroda, Masahiro Kurosaka

**Affiliations:** 1 Department of Orthopedic Surgery, Kobe University Graduate School of Medicine, Kobe, Japan; 2 Division of Rehabilitation Medicine, Kobe University Graduate School of Medicine, Kobe, Japan; University of Louisville School of Medicine, UNITED STATES

## Abstract

The potential relationship between cell cycle checkpoint control and tissue regeneration has been indicated. Despite considerable research being focused on the relationship between p21 and myogenesis, p21 function in skeletal muscle regeneration remains unclear. To clarify this, muscle injury model was recreated by intramuscular injection of bupivacaine hydrochloride in the soleus of p21 knockout (KO) mice and wild type (WT) mice. The mice were sacrificed at 3, 14, and 28 days post-operation. The results of hematoxylin-eosin staining and immunofluorescence of muscle membrane indicated that muscle regeneration was delayed in p21 KO mice. *Cyclin D1* mRNA expression and both Ki-67 and PCNA immunohistochemistry suggested that p21 deficiency increased cell cycle and muscle cell proliferation. F4/80 immunohistochemistry also suggested the increase of immune response in p21 KO mice. On the other hand, both the mRNA expression and western blot analysis of *MyoD*, *myogenin*, and *Pax7* indicated that muscular differentiation was delayed in p21KO mice. Considering these results, we confirmed that muscle injury causes an increase in cell proliferation. However, muscle differentiation in p21 KO mice was inhibited due to the low expression of muscular synthesis genes, leading to a delay in the muscular regeneration. Thus, we conclude that p21 plays an important role in the *in vivo* healing process in muscular injury.

## Introduction

Sports-related muscle injuries are among the most common soft tissue injuries [1,2]. In most cases, they are caused by sudden overstretching of the contracted muscle, leading to muscle fiber damage [3,4]. Such injuries do not usually necessitate surgery, a majority of these can be treated with conservative therapy [5]. In cases where muscle injury is judged as not being a severe pathological condition, the therapy duration might even be reduced. This reduction in treatment period may lead to recurrence and intensification of damage. Furthermore, injury can cause problems that interfere with day-to-day activities. The degree of muscular damage also considerably affects the individual’s return to sports, mainly in injuries affecting the lower leg. Therefore, it is essential to heal muscle injuries quickly and completely. Thus far, considerable research has been conducted on approaches used to expedite muscle recovery [6–9]. While it is important to consider different approaches to muscle recovery these options for muscular recovery, a more effective and attractive approach would involve the use of a gene-specific treatment, which could even affect a quicker, complete recovery.

The initial phase of muscle repair is characterized by necrosis of the damaged tissue and activation of an inflammatory response. Skeletal muscle development is a highly regulated process, involving the specification of mesoderm-derived precursors into myoblasts followed by the differentiation and fusion of these cells into multinucleated myotubes [10]. *Pax3* (paired box transcription factor family) expression in precursor cells contributes to myogenic cell expansion. Induction by *Myf5* (myogenic factor) and/or *MyoD* (myogenic regulatory factor [MRF] family) causes mesodermal somitic cells to be committed to the myogenic lineage (myoblasts). The upregulation of secondary MRFs (*myogenin* and *MRF4*) induces the terminal differentiation of myoblasts into myocytes. Finally, myocyte fusion generates multinucleated myofibers. During the late phase of embryonic myogenesis, a distinct population of myoblasts, derived from satellite cells, fuses to existing myofibers and thus enables myofiber growth. Some satellite cells remain closely associated with myofibers in a quiescent, undifferentiated state. While the embryonic origin of satellite cells is yet to be determined, *Pax7* expression is known to be essential for the specification and expansion of the satellite cell population.

p21 (cyclin-dependent kinase inhibitor 1) suppresses abnormal DNA increase induced by external stress, by regulating the cell cycle. p21 protein plays an essential role in G1 checkpoint by binding to and inhibiting the activity of cyclin-dependent kinase (CDK) 2, CDK1, and CDK4/6 enzyme complexes, thereby acting as a cell cycle regulator at G1 and S phases [11,12]. Bedelbaeva et al. reported that the wound healing capacity improved after ear hole punching in p21 knockout mice [13]. Olive et al. observed that p21 plays an important role in the modulation of vascular remodeling after arterial injury [14]. These reports indicate the potential relationship between cell cycle checkpoint control and tissue regeneration. Despite considerable research being focused on the relationship between p21 and myogenesis [15–17], the mechanism of p21 function in myogenic differentiation remains unclear. The aim of this study was to confirm the influence of p21 in the muscle damage recovery, and to investigate p21 function in skeletal muscle regeneration after injury.

## Materials and Methods

### Mouse Strains and Breeding

This study was carried out in strict accordance with the recommendations in the Guide for the Care and Use of Laboratory Animals of the National Institute of Health. All procedures were approved by the Animal Studies Committee of Kobe University, Japan [Permit Number: P131104]. P21 mice (KO) mice (B6.129S6(Cg)-*Cdkn1a* ^*tm1Led*^/J) were obtained from The Jackson Laboratory (Bar Harbor, Maine, USA) and bred in our animal facility. All mice were housed in cages under pathogen-free conditions and were allowed free access to water and food. P21 KO and wild-type (WT) mice (C57BL/6J) were crossed to generate heterozygous mice. The heterozygous mice were then crossed to obtain p21KO and WT mice as littermates.

### Mouse Genotyping

Genotypes were confirmed by polymerase chain reaction analysis of DNA obtained from mouse tails. Genomic DNA was extracted using the DNeasy Blood & Tissue Kit (Qiagen, Valencia, California, USA) in accordance with the manufacturer’s protocol. p21 deletion was confirmed by the presence of a 447 base pair fragment unique to the mutant genotype, amplified with p21-specific forward primer (GTT GTC CTC GCC CTC ATC TA) and mutant reverse primer (CTG TCC ATC TGC ACG AGA CTA) (sequences provided by The Jackson Laboratory). Wild-type alleles (240-bp fragment) were amplified with the wild-type reverse primer (GCC TAT GTT GGG AAA CCA GA) and p21-specific forward primer. DNA amplification was performed under the following PCR conditions: 94°C for 5 min; followed by 40 cycles of 94°C for 30 s, 55°C for 30 s, and 72°C for 30 s; and a final extension at 72°C for 2 min.

### Bupivacaine-induced Muscle Injury Model

p21 KO mice and WT mice (10 weeks, n = 9 for each time point) were anesthetized by an intraperitoneal injection of pentobarbital (50 mg/kg). The muscle injury model was recreated based on previously tested protocols; muscle damage was induced by intramuscular injection of 0.1 mL of 0.5% bupivacaine hydrochloride (AstraZeneca, London, UK) in the soleus of mice [18,19], using a 27 gauge needle. The mice were anesthetized by an intraperitoneal injection of pentobarbital (50 mg/kg) and sacrificed by cervical dislocation at 3, 14, and 28 days post-operation with all effort to minimize suffering. The muscles of non-operative mice were also obtained as control. After weighing the total body and muscle samples in each mouse, the samples were fixed in 4% paraformaldehyde buffered with phosphate-buffered saline, decalcified with 10% formic acid, and embedded in paraffin. Axial histological sections (6 μm thick) were cut with a microtome, and stained with hematoxylin-eosin (HE). Tissue sections were then subjected to immunofluorescence analysis.

### Immunofluorescence

De-paraffinized sections were digested with proteinase (Dako Retrieval Solution Ready-to-Use; Dako, Glostrup, Denmark) for 20 min, and the sections were treated overnight at 4°C with the antibodies in Can Get Signal immunostain Solution A (Toyobo, Osaka, Japan); goat anti-mouse Laminin α-1 polyclonal antibody (1:50 dilution; Santa Cruz Biotechnology, Santa Cruz, California, USA) and rabbit anti-mouse Dystrophin polyclonal antibody (1:100 dilution; Santa Cruz Biotechnology). The sections were then incubated with the secondary antibodies; chicken anti-goat immunoglobulin Alexa Fluor 488 (1:100 dilution; Life Technologies, Carlsbad, California, USA), and chicken anti-rabbit immunoglobulin Alexa Fluor 594 (1:100 dilution; Life Technologies) for 60 min at room temperature. The nucleus was stained with DAPI, and images were obtained using a BZ-X700 microscope (Keyence, Osaka, Japan).

### Immunohistochemistry

De-paraffinized sections obtained from control mice and post-operative mice at day 3 were digested with proteinase (Dako Retrieval Solution Ready-to-Use; Dako) for 20 min and treated with 3% hydrogen peroxide (Wako Pure Chemical Industries, Osaka, Japan) to block endogenous peroxidase activity. In this study, we examined the expression of Ki-67 to evaluate cell proliferation. Because Ki-67 is present during active phases of the cell cycle, but absent in resting cells, it is often used as an excellent marker for cell proliferation [20]. Furthermore, we examined the expression of PCNA and F4/80 in the same sections to evaluate the healing process with regard to the immune response and infiltration of other cells among the proliferating cells. The PCNA gene encodes an essential DNA replication accessory protein and plays a central role at the replication fork, recruiting and retaining many of the enzymes required for DNA replication and repair [21]. F4/80 is expressed at high levels on the surface of various macrophages [22]. Macrophages play a critical role in inflammation with three major functions—antigen presentation, phagocytosis, and immunomodulation—through the production of various cytokines and growth factors [23]. Tissue sections were treated overnight at 4°C with a rabbit anti-mouse Ki-67 monoclonal antibody (1:400 dilution; Cell Signaling Technology), rabbit anti-mouse PCNA polyclonal antibody (1:500 dilution; abcam, Cambridge, UK), and rat anti-mouse F4/80 monoclonal antibody (1:100 dilution; AbD Serotec, Kidlington, UK) in Can Get Signal immunostain solution A (Toyobo). Subsequently, tissue sections were treated with a peroxidase-labeled anti-rabbit immunoglobulin antibody [Histofine Simple Stain Mouse MAX PO (R); Nichirei Bioscience] and a peroxidase-labeled anti-rat immunoglobulin antibody [Histofine Simple Stain Mouse MAX PO (Rat); Nichirei Bioscience] at room temperature for 30 min. The signal was developed as a brown reaction product using the peroxidase substrate, 3, 3’-diaminobenzidine, (Histofine Simple Stain DAB Solution, Nichirei Bioscience) and the sections were examined using a BZ-X700 microscope (Keyence).

### RNA Isolation and Quantitative Reverse Transcription-Polymerase Chain Reaction (RT-PCR)

Total RNA was extracted from all samples using TRIzol reagent (Invitrogen, Carlsbad, California, USA), followed by RNeasy (Qiagen), treatment according to the manufacturer’s protocol. Complementary DNA (cDNA) was obtained by reverse-transcription of total RNA with SuperScript II reverse transcriptase (Invitrogen). Quantitative RT-PCR was performed with 20 μL of reaction mixture containing SYBR Green PCR Master Mix (Applied Biosystems, Foster City, California, USA) and primers for *Cyclin D1*, *MyoD*, *myogenin*, and *Pax7*, using the ABI Prism V.7700 sequence detection system (Applied Biosystems). The comparative Ct (threshold cycle) method was used to evaluate mRNA expression levels of all genes in p21KO and WT mice.

### Primers for Quantitative RT-PCR

Primer sequences used were as follows: Cyclin D1 (sense 5′-GGG GAC AAC TCT TAA GTC TCA C-3-; anti-sense 5′-CCA ATA AAA GAC CAA TCT CTC-3′), MyoD (sense 5′-AGT AGA GAA GTG TGC GTG CT-3′; anti-sense 5′-ACG ACT TCT ATG ATG ATC CG-3′), Myogenin (sense 5′-CCA ACC CAG GAG ATC ATT TG-3′; anti-sense 5′-ACG ATG GAC GTA AGG GAG TG-3′), and *Pax7* (sense 5′-GCC AAG AGG TTT ATC CAG CC-3′; anti-sense 5′-AGA GGG GTG GAC ACT TCC AG-3′).

### Western Blotting Assay

Muscular tissues obtained from control mice and post-operative mice at day 3 were lysed in hypotonic lysis buffer (25 mM Tris, 1% NP-40, 150 mM NaCl, 1.5 mM EGTA) supplemented with protease and phosphatase inhibitor mix (Roche Diagnostics, Basel, Switzerland). Protein concentration was quantified using the BCA Protein Assay Kit (Thermo Scientific, Rockford, IL, USA) and standardized by dilution with hypotonic buffer. Each sample was mixed with sample buffer, electrophoresed on a 7.5–15% polyacrylamide gradient gel (Biocraft, Tokyo, Japan) and transblotted electrically onto the blotting membrane (GE Healthcare, Buckinghamshire, UK). Membranes were incubated overnight at 4°C with the following antibodies in Can Get Signal Solution 1 (Toyobo): anti-MyoD antibody (1:200 dilution; Santa Cruz Biotechnology), anti-Myogenin antibody (1:1000 dilution; Abnova, Taipei, Taiwan), anti-Pax 7 antibody (1:1000 dilution; abcam), and anti-mouse α-tubulin antibody (1:5000 dilution; Sigma-Aldrich, St. Louis, MO, USA). Horseradish peroxidase (HRP)-conjugated donkey anti-rabbit IgG antibody (1:2000 dilution; GE Healthcare Bio-Sciences, Piscataway, NJ, USA) was used as the secondary antibody and the signals were visualized with the ECL Plus western blotting detection system reagent (GE Healthcare) using a Chemilumino Analyzer LAS-3000 mini (FUJI FILM, Tokyo, Japan).

### Statistical Analysis

Statistical analysis was performed using the SPSS software package (IBM, Chicago, Illinois, USA). The differences in the ratios of muscle weight to body weight between the different groups at each time point were analyzed using the Mann Whitney U-test. The differences in the percentages of Ki-67-, PCNA-, and F4/80-positive cells between WT and p21KO groups at each time point were also analyzed by the Mann Whitney U-test. The relative expression ratios in immunofluorescence experiments were quantified using selective coloring in Adobe Photoshop (CS6; Adobe Systems, San Jose, CA, USA) [24]. All values are presented as mean ± standard error. Results with p < 0.05 were considered statistically significant.

## Results

### Muscle Weight

The average muscle weights of WT and p21 KO groups at each time point are indicated in Table 1. No significant differences were observed between the two groups with regard to the ratio of muscular and body weight at control, 3, and 28 days after injury. However, the ratio in p21KO mice 14 days after injury was significantly reduced compared to that of WT mice. This result indicated that muscle regeneration was impaired in p21KO mice.

**Table 1:** Comparison of muscle weight/body weight.

	Muscle weight (mg)		Ratio of muscle weight / body weight		
Day	WT	p21KO	WT	p21KO	P value
control	0.14 ± 0.00	0.14 ± 0.01	5.57 ± 0.08	6.25 ± 0.42	0.17
3	0.16 ± 0.01	0.19 ± 0.02	6.44 ± 0.35	6.91 ± 0.36	0.38
14	0.15 ± 0.00	0.13 ± 0.01	5.70 ± 0.09	5.36 ± 0.05	0.01
28	0.20 ± 0.01	0.16 ± 0.01	6.89 ± 0.24	6.41 ± 0.84	0.57

± Standard error.

### HE Staining

**Fig 1:**
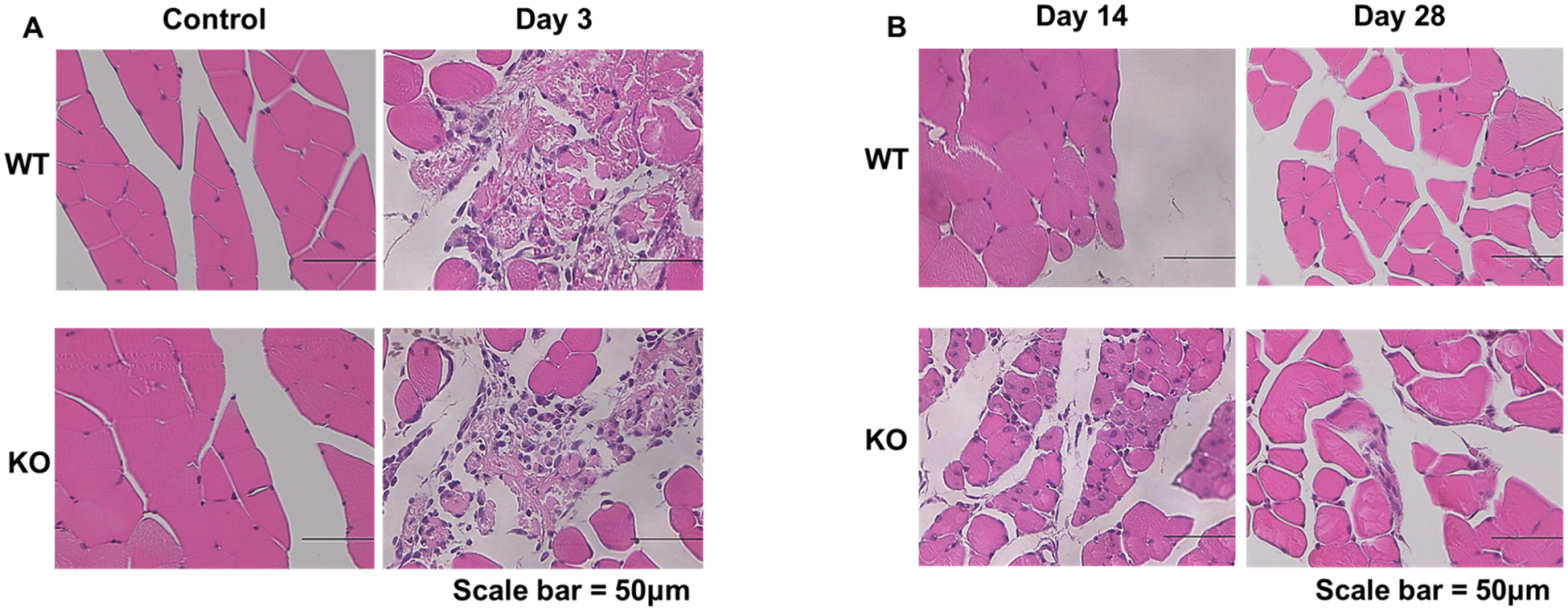
Histological analysis of muscle tissues control, 3, 14, and 28 days post-operation hematoxylin-eosin (HE) staining. WT: wild type, KO: knockout. (A) Muscle tissues at control and 3 days post-operation, (B) Muscle tissues at 14 and 28 days post-operation. (Scale bar = 50μm). (A and B) The injured muscle was almost recovered 14 days after injury in WT group, but not in the p21 KO group.

*In vivo* muscle regeneration was analyzed by histological analysis of muscular tissues at control, 3, 14, and 28 days post-operation. HE staining revealed that muscle regeneration in p21KO mice was significantly impaired compared to WT mice (Fig 1). The inflammatory response, such as inflammatory cell infiltration, was observed 3 days after injury in both groups (Fig 1A). The injured muscle was almost recovered 14 days after injury in WT group, but not in the p21 KO group (Fig 1B). The injured muscle was almost recovered 28 days after injury in p21 KO group. These results indicated that muscle regeneration was delayed in p21 KO mice.

### Immunofluorescence Evaluation of Muscle Basement Membrane and Plasma Membrane

**Fig 2:**
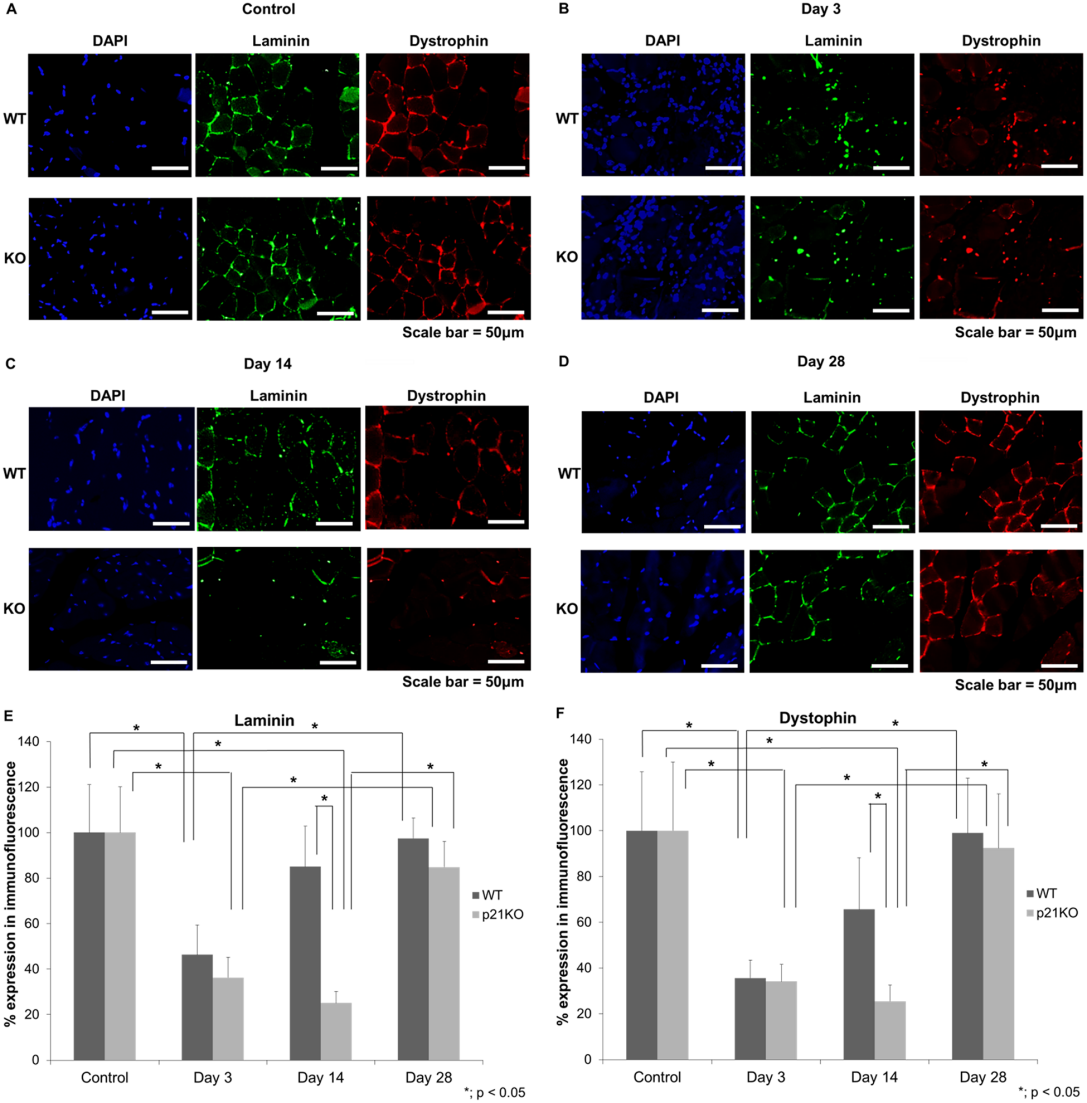
Immunofluorescence analysis of muscle basement membrane and plasma membrane. The expression of DAPI, laminin, and dystrophin was examined in wild-type (WT; upper panel) and p21 knockout (KO; lower panel) mice. (A) Expression in control, (B) Expression in 3 days, (C) Expression in 14 days, and (D) Expression in 28 days post-operation. (Scale bar = 50μm). (E) Quantification of laminin expression, (F) Quantification of dystrophin expression. (A-F) The injured membrane structure was almost repaired 14 days after injury in WT group, but not in the p21 KO group (p < 0.05).

The muscle structure in each recovery period was elucidated and compared using two membrane-associated genes. A mutation to *Dystrophin* gene that encodes dystrophin causes Duchenne muscular dystrophy, and has been suggested as being a major component of the sub-sarcolemmal cytoskeleton involved in maintaining the integrity of the myofiber plasma membrane [25,26]. Laminins are essential structural glycoproteins localized in the basement membranes that influence cell proliferation and tissue architecture [27,28]. Immunofluorescence analysis using laminin and dystrophin revealed that muscle regeneration in p21KO mice was significantly impaired compared to WT mice (Fig 2). Membranous structure was not recovered 3 days after injury in WT and p21KO groups (Fig 2B). The injured membrane structure was almost repaired 14 days after injury in WT group (Fig 2C). These membranes were almost completely recovered 28 days after injury in p21KO groups (Fig 2D). Quantification of the relative expression ratios of laminin and dystrophin, detected by immunofluorescence, also revealed that these membrane-associated genes showed the lowest expressions 3 days after injury in both the groups (Fig 2E and 2F). Furthermore, laminin and dystrophin expression was significantly increased 14 days after injury in the WT group than that in the p21KO group. These results indicated that muscle membrane regeneration was also delayed in p21 KO mice.

### mRNA Expression of *Cyclin D1*

**Fig 3:**
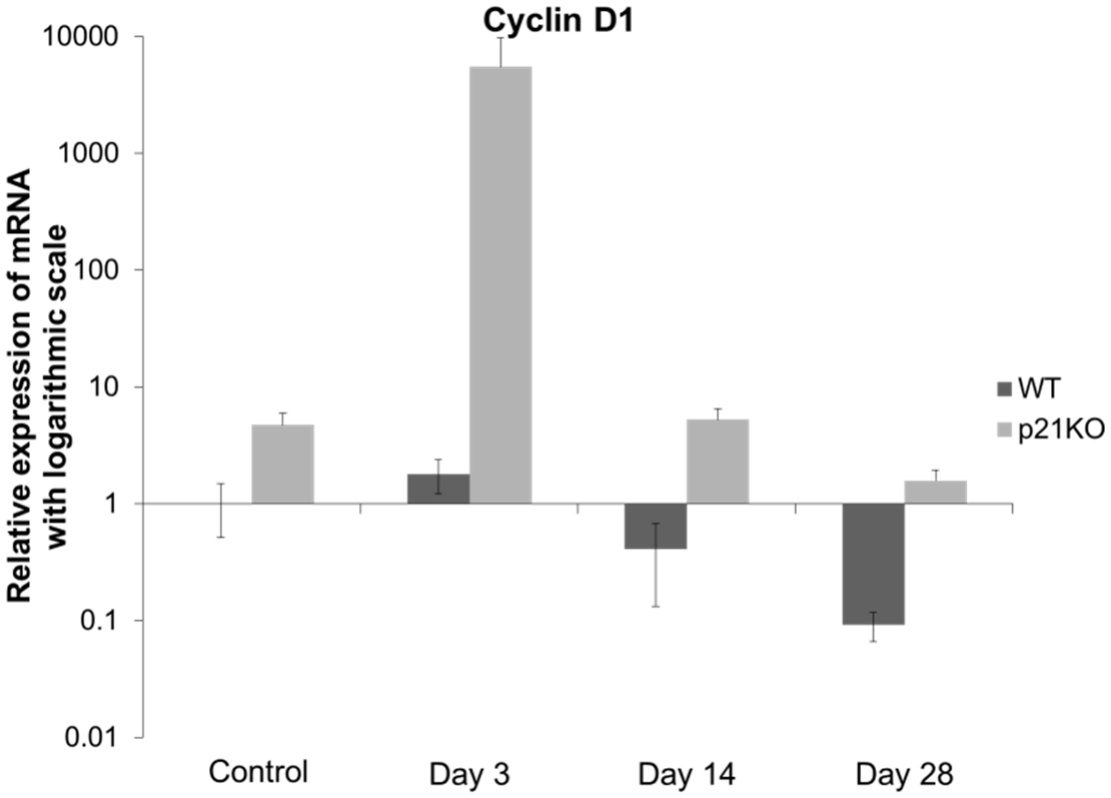
Relative expression of *Cyclin D1* mRNA (expressed in the logarithmic scale). WT: wild type, KO: knockout. *Cyclin D1* expression levels were significantly higher in p21 KO group than in WT group, through the 28 days post-injury.

*Cyclin D1* expression levels in no- operative WT mice at 0-day was used as the control. *Cyclin D1* expression increased 3 days after injury in both groups. *Cyclin D1* expression levels were significantly higher in p21 KO group than in WT group, through the 28 days post-injury. These results suggested that p21 deficiency increased cell cycle and muscle cell proliferation (Fig 3).

### Ki-67 Immunohistochemical Analysis

**Fig 4:**
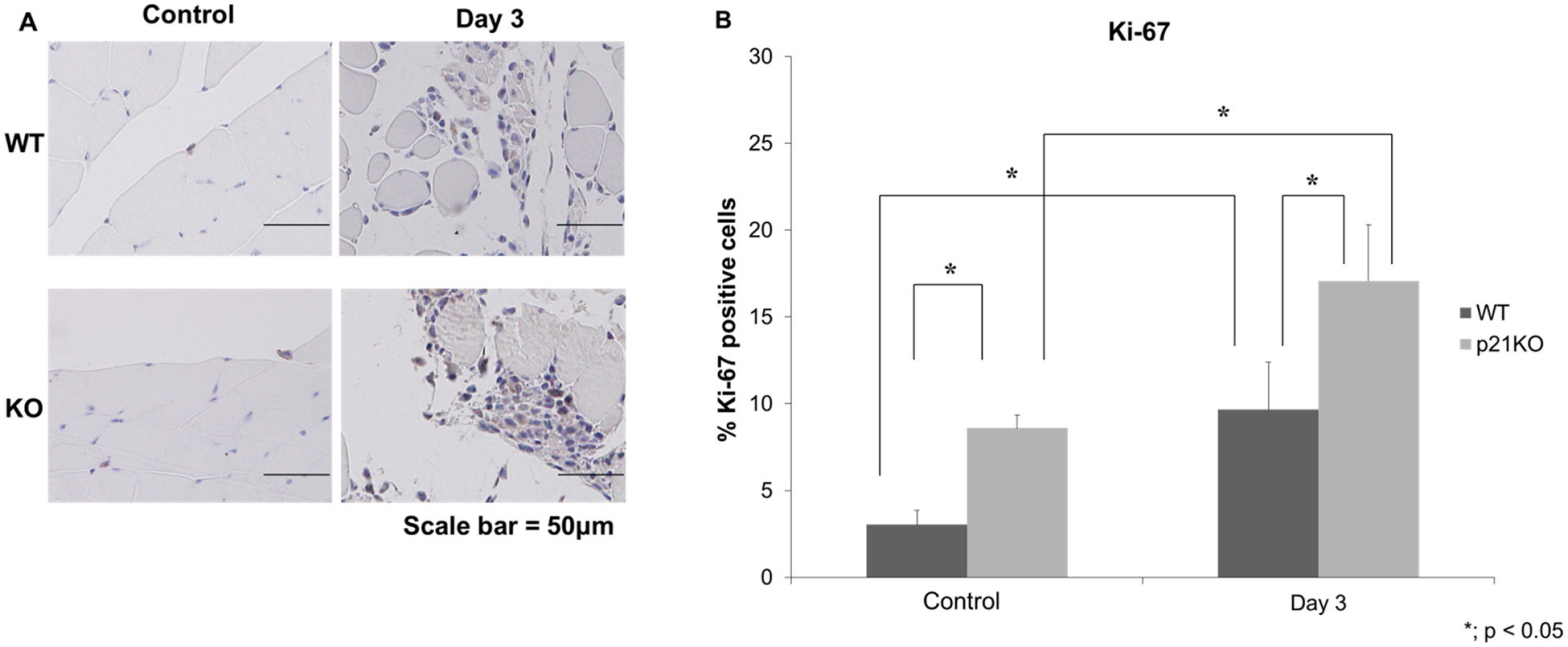
Ki-67 expression after muscular injury. (A) Immunohistochemistry of Ki-67, (B) Quantitative analysis of Ki-67. (A and B) Ki-67 expression levels in WT and p21KO mice were significantly different at control and 3 days after injury (p < 0.05). Ki-67 expression in p21KO mice at 3 days after injury was the highest (p < 0.05).

Ki-67 expression levels were significantly different at control and at 3 days after injury between WT and p21KO mice (Fig 4A and 4B). Furthermore, the number of Ki-67 positive cells also significantly increased at control and at 3 days after injury in each groups. These results indicated that p21 deficiency increased cell proliferation postoperatively.

### PCNA and F4/80 Immunohistochemical Analysis

**Fig 5:**
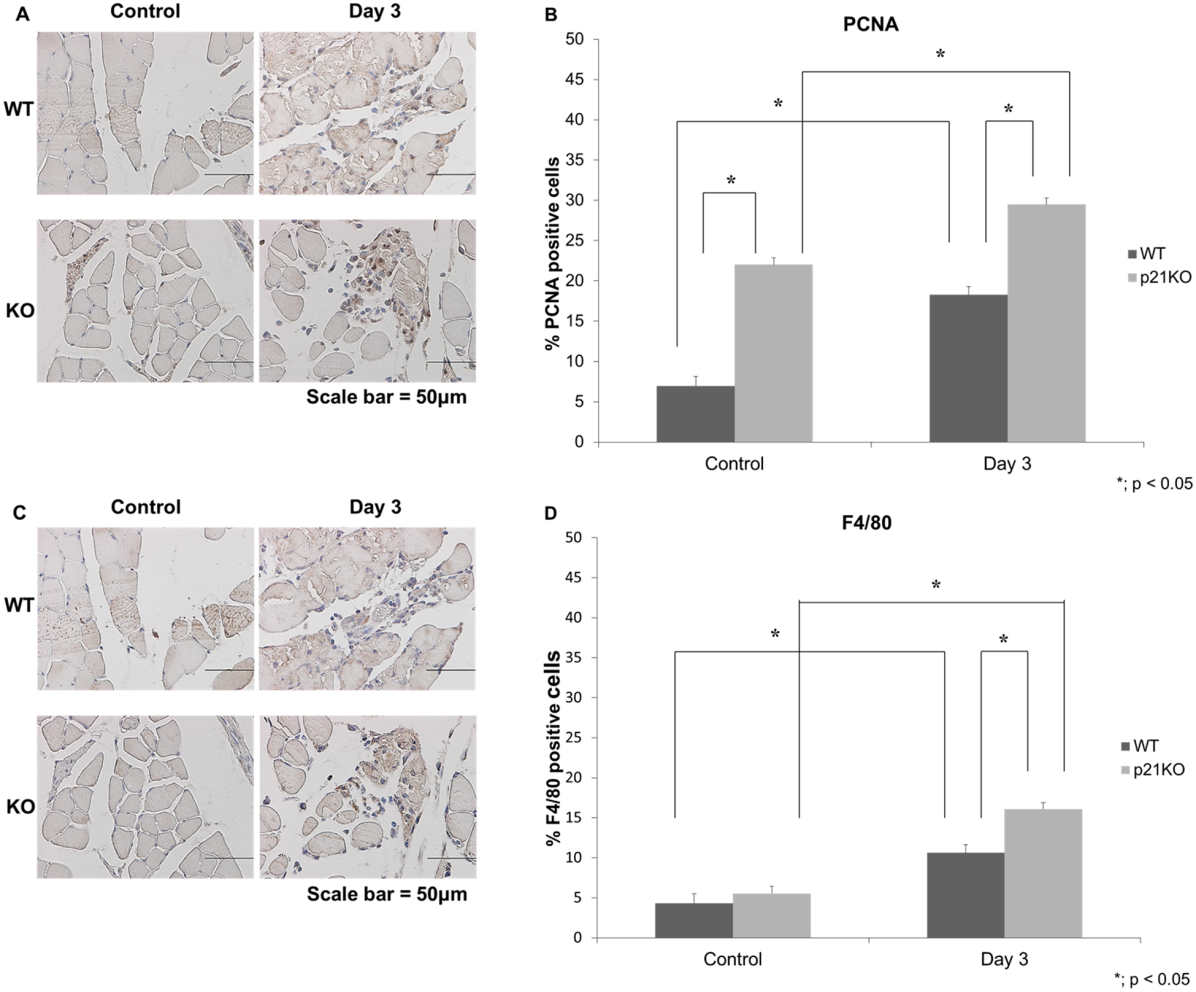
PCNA and F4/80 immunohistochemical expression after muscular injury. WT: wild type, KO: knockout. (A) Immunohistochemistry of PCNA. (Scale bar = 50μm), (B) Quantitative analysis of PCNA-positive cells, (C) Immunohistochemistry of F4/80. (Scale bar = 50μm), (D) Quantitative analysis of F4/80-positive cells. (B and D) Both PCNA and F4/80 expression in p21KO mice at 3 days after injury were the highest (p < 0.05).

The expression of PCNA was significantly higher at 3 days after injury than that observed at control in both groups. The expression levels of PCNA were significantly higher in p21KO than in WT mice 3 days after injury (Fig 5A and 5B). In the same section, the expression of F4/80 was the highest in p21KO mice at 3 days after injury (Fig 5C and 5D). These results indicated that cell proliferation and the inflammatory response were increased in p21 KO mice. When we focused on the difference in the PCNA- and F4/80-positive cells on the same field, the PCNA- and F4/80-positive cells were not identical (Fig 5A and 5C). These results suggested that PCNA-positive and F4/80-negative cells represented myogenic progenitor cells.

### mRNA expression of Myogenic Differentiation-Related Genes

**Fig 6:**
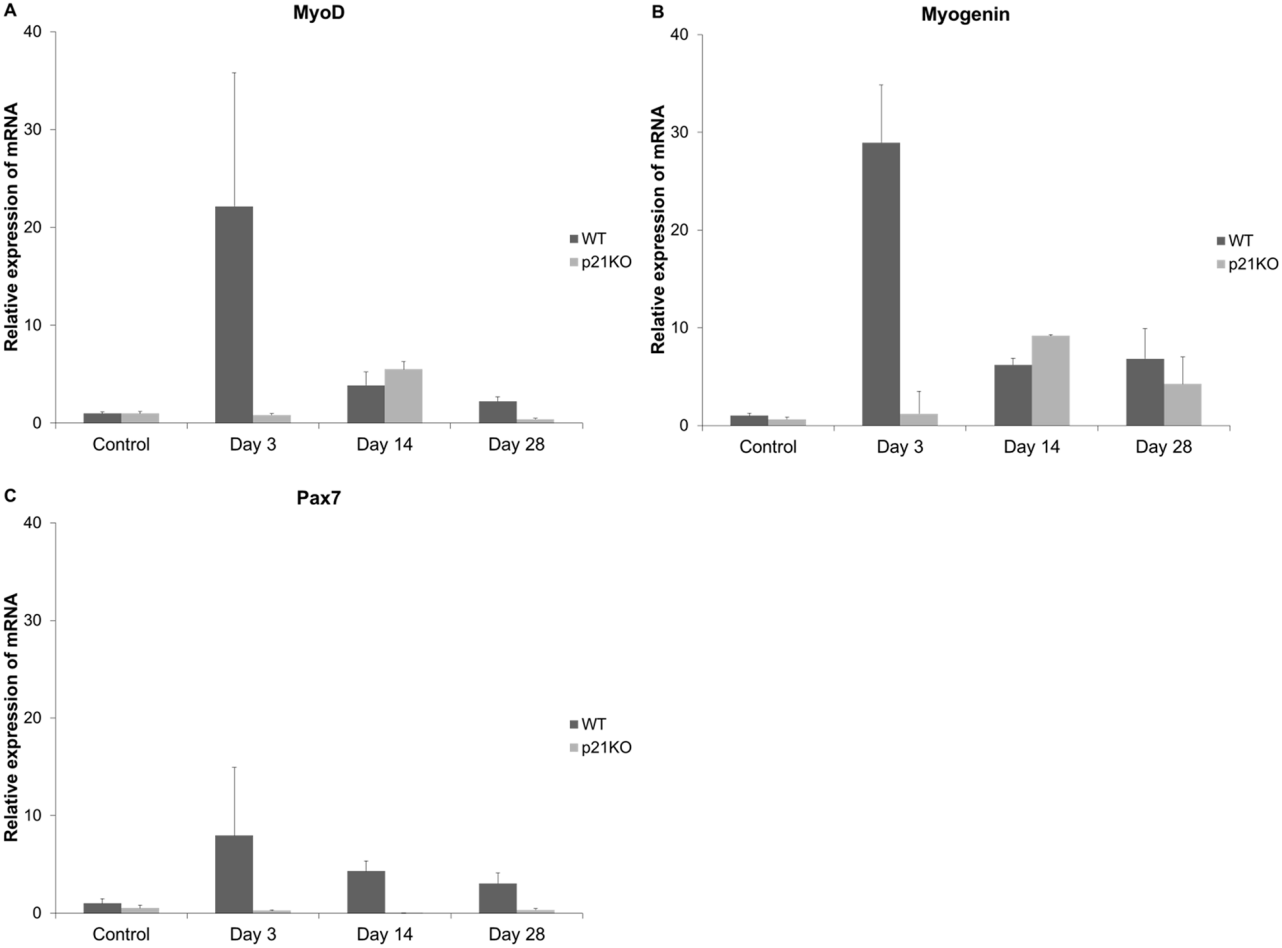
mRNA expression profiles of myogenic markers. The relative mRNA expression of (A) *MyoD*, (B) *myogenin*, and (C) *Pax7* was determined in wild-type (WT) and p21 knockout (KO) mice using the relative gene expression level at 0 day post-operation as control. (A-C) The mRNA expression of *MyoD*, *myogenin*, and *Pax7* peaked on day 3 in WT mice and on day 14 for *MyoD* and *myogenin* in KO mice. The peaks for WT were observed to be stronger than those for KO mice.

The peak expression levels of *MyoD* and *myogenin* were observed 3 days after injury in WT group. However, the peak levels for gene expression in p21 KO group were only observed 14 days post-injury (Fig 6A and 6B). In addition, the peak expression levels of *MyoD* and *myogenin* were much lower in the p21KO group. The peak expression level of *Pax7* was at 3 days after injury in WT group (Fig 6A and 6B). However, *Pax7* expression level did not increase in the p21KO group (Fig 6C). These results indicated that muscular differentiation was impaired and delayed in p21KO mice.

### Western blotting Analysis of Myogenic Differentiation-Related Proteins Obtained from WT and p21KO Muscular Injury Mouse Models

**Fig 7:**
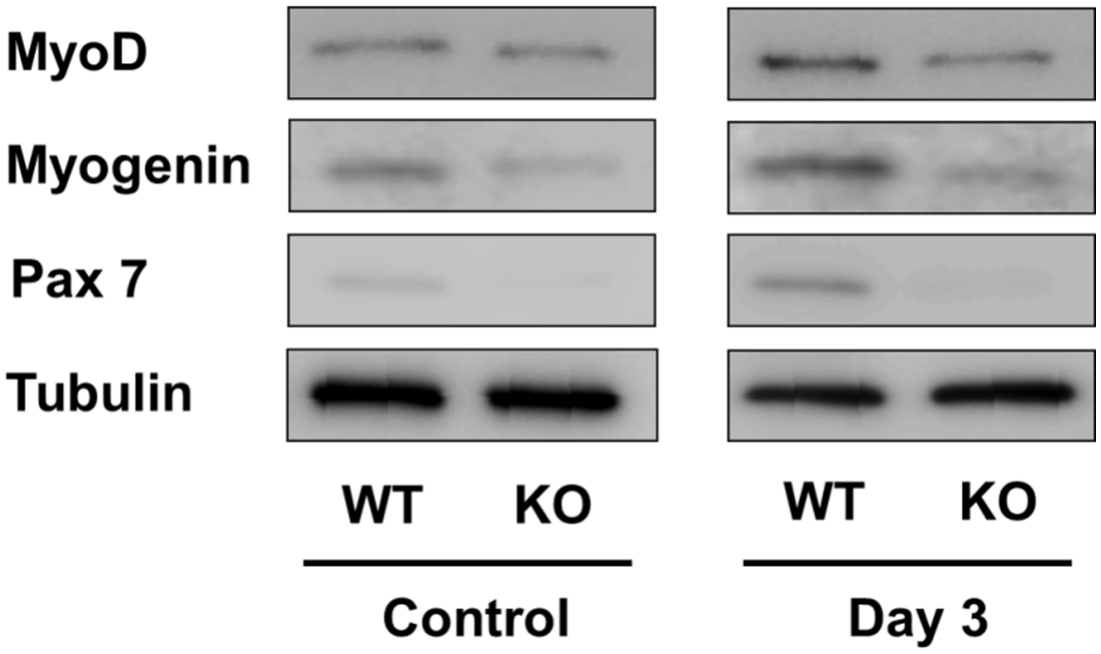
Western blot analysis of myogenic differentiation-related proteins. WT: wild type, KO: knockout. The expression levels of *MyoD*, *myogenin*, and *Pax7* was increased 3 days after injury in WT mice, but not in p21KO mice.

Western blotting analysis revealed that the expression levels of MyoD, myogenin, and Pax7 at 3 days after injury were increased in the WT group (Fig 7). However, no change was observed in the p21KO group. These results were consistent with the results of mRNA expression evaluated by quantitative RT-PCR.

## Discussion

In this study, we investigated the role of p21 in muscle regeneration. The histological findings and experiments conducted to determine weight of the injured muscle demonstrated that the injured muscle showed almost full recovery at 14 days in WT mice, which was consistent with another muscle injury model [29]. However, no recovery patterns were observed at 14 days in p21 KO mice. Immunofluorescence staining also showed that recovery of the muscular membranous structure was significantly delayed in p21KO mice compared to WT mice. These results indicated that muscle regeneration was delayed in p21 KO mice.

From the cell cycle point of view, increased mRNA expression of *cyclin D1* was observed in p21KO mice compared to WT mice. Additionally, the most severe muscle damage was observed 3 days post-injury, a finding consistent with a previous report [30]. The main function of p21 is as a negative regulator of the G1/S transition, inducing “G1 arrest.” Therefore, cyclin D1 is best suited for the evaluation of cell cycle progression at G1 phase [31]. Cyclin D1 forms complexes with CDK4 or CDK6, whose activity is required for cell cycle G1/S transition. Our results indicate that cell proliferation in response to muscle injury was higher in p21KO mice than in WT mice. Furthermore, Ki-67 expression was increased in p21KO mice compared to WT mice as measured by immunohistochemistry. Ki-67 is a well-characterized and extensively used proliferation marker [20]. After DNA damage, repair synthesis is completely dependent on PCNA, which interacts with many accessory proteins and coordinates multiple enzymatic activities [21,32]. We demonstrated the increase in PCNA expression in p21 KO mice and revealed that cell proliferation was increased in p21 KO mice. Marvers et al. reported increased numbers of macrophages and severe articular destruction in p21 KO mice [33]. We demonstrated that the increase in F4/80 expression indicated an increase in the immune and inflammatory response in p21 KO mice. Further, the PCNA- and F4/80-positive cells were not identical. These results indicated that myogenic progenitor cells were increased in p21 KO mice after injury.

Thus, these results suggest that cell proliferation is promoted in the p21KO mice injury model. Furthermore, we evaluated cell proliferation by using a p21 siRNA and water-soluble tetrazolium salt assay (S1 and S2 Figs). The results also supported our *in vivo* findings.

A number of studies have been conducted on the association of *MyoD* and *Myogenin* with p21 in myogenesis [15,16]. Cell cycle withdrawal by proliferating myoblast is a key facet of muscle differentiation [34]. p21 transcription has been reported to be induced by MyoD and plays an important role in coupling irreversible withdrawal from the cell cycle by differentiating during myogenesis [15]. These genes have demonstrated high mRNA expression levels during the early phase of injury [35]. This observation is consistent with the results observed in WT mice. However, a delayed and decreased expression of *MyoD* and *Myogenin* was observed in p21KO mice. Western blot analysis also confirmed MyoD and myogenin expression when both groups were compared. These findings indicate that p21KO affect the differentiation of myoblasts, myotubes, and myofibers in muscular regeneration.

Muscular satellite cells, which exist at the earliest stage of myogenesis, must be activated in order to fuse with each other or with existing muscular cells to promote muscle regeneration [36]. When the basement membrane is damaged in muscle injury, the satellite cells that are attached to the basement membrane are also damaged, causing a delay in muscle regeneration. In other words, if the integrity of the basement membrane remains uncompromised, the scaffold for muscular regeneration will remain. Thus, muscle satellite cells play an important role in muscular regeneration[35]. Immunofluorescence analysis showed that the expression of laminin was decreased in p21KO mice. This finding indicates morphological changes had occurred in the basement membrane. Further, the expression levels of *Pax7* did not show any significant change throughout 28 days of analysis. Therefore, we concluded that the muscular regeneration process in basal membrane level, associated with *Pax7* expression and the corresponding protein activity, was also impaired in p21KO mice. Considering these results, we confirmed that muscle injury causes an increase in cell proliferation. However, muscle differentiation in p21 KO mice was inhibited due to the low expression of muscular synthesis genes that never reach the WT values, leading to a delay in the muscular regeneration.

In spite of these results, the completion of muscular regeneration seemed to be eventually obtained in p21KO mice. Recent studies revealed the close relationship between RNA-binding proteins such as the human antigen R (HuR) and the KH-type splicing regulatory protein (KSRP) and gene regulation in myogenesis [34,37]. Additionally, several signaling pathways have also been identified in the past such as p38MAPK, Wnt, and mTOR [37–39]. Therefore, although p21 deletion affects various muscular recovery stages based on our results, there is some redundancy in muscular regeneration. Furthermore, p21 deletion also has the possibility of being compensated by other cell cycle regulator [40]. Therefore, it is suggested that various complicated mechanisms control the muscular regeneration in adult mice.

In conclusion, our study revealed that cell proliferation was increased, but muscular differentiation was decreased in p21KO mice. Thus, we conclude that p21 plays an important role in the *in vivo* healing process in muscular injury.

## Supporting Information

S1 Fig p21mRNA expression in control siRNA or p21 siRNA transfected C2C12 cells. In control siRNA, p21 mRNA expression was significantly increased in differentiation medium (p < 0.05).
(TIF)

S2 Fig p21 knockdown increased cell proliferation. Cell proliferation significantly increased in both media in p21 siRNA transfected C2C12 cells (p < 0.05).
(TIF)

S1 File Supplementary Manuscript. (DOCX)
