# Supplementary figures and images for: P21 Deficiency Delays Regeneration of Skeletal Muscular Tissue
Source: PLoS One. 2015 May 5;10(5):e0125765. doi: 10.1371/journal.pone.0125765 (PMC4420284; doi:10.1371/journal.pone.0125765)

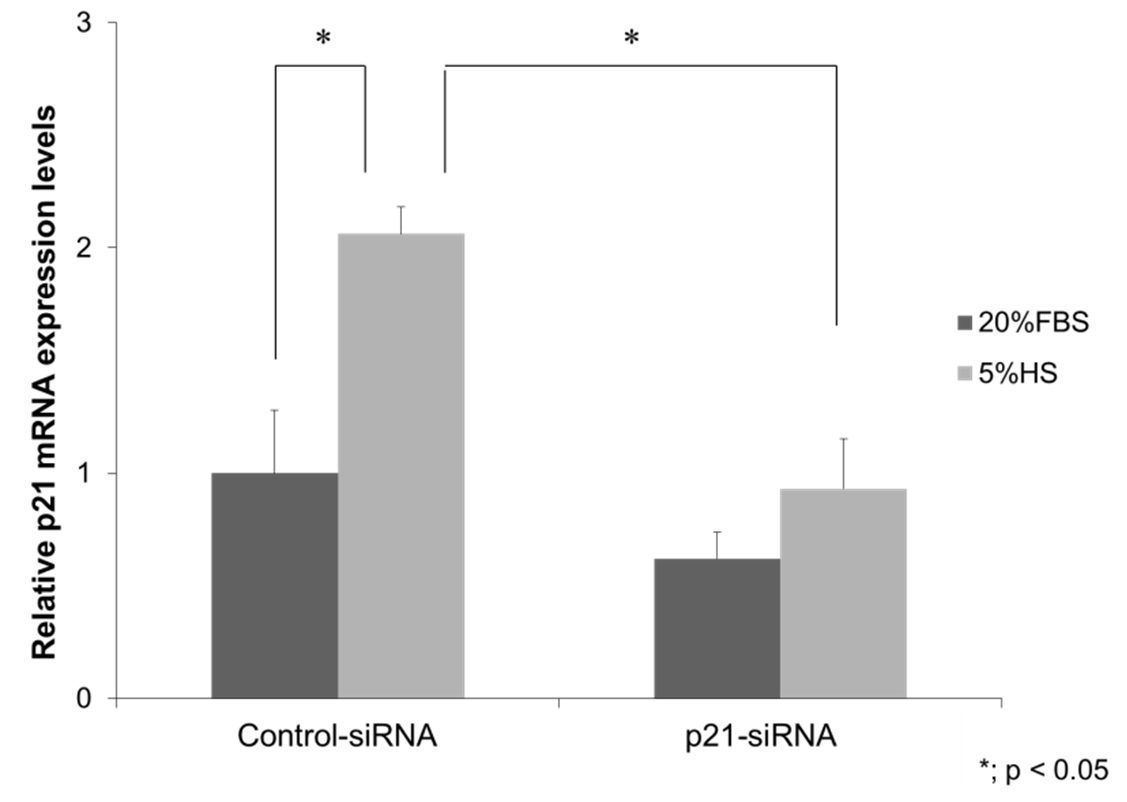

Supplement: S1 Fig — In control siRNA, p21 mRNA expression was significantly increased in differentiation medium (p < 0.05). (TIF) [file pone.0125765.s001.tif]

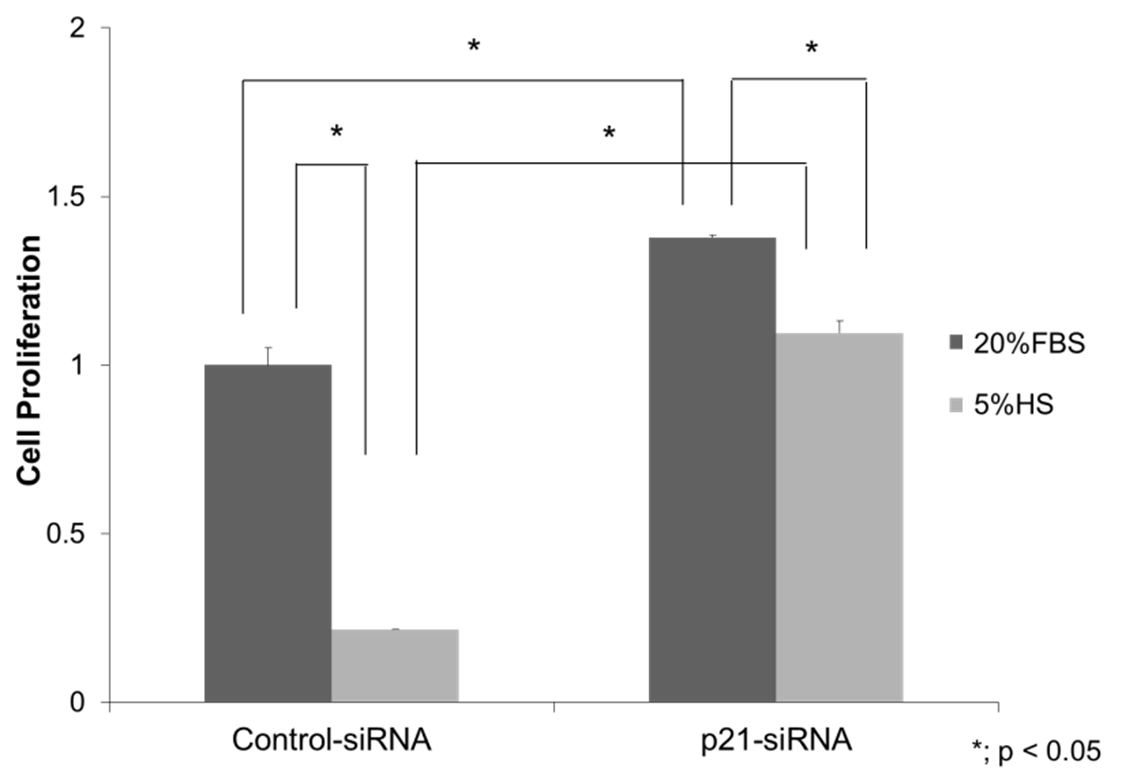

Supplement: S2 Fig — Cell proliferation significantly increased in both media in p21 siRNA transfected C2C12 cells (p < 0.05). (TIF) [file pone.0125765.s002.tif]
