# Supplementary material for: P21 Deficiency Delays Regeneration of Skeletal Muscular Tissue
Source: PLoS One. 2015 May 5;10(5):e0125765. doi: 10.1371/journal.pone.0125765 (PMC4420284; doi:10.1371/journal.pone.0125765)
Supplement: S1 File — (DOCX) [file pone.0125765.s003.docx]

**Material and Methods**

**Mouse myoblast cell lines**

The mouse myoblast cell line, C2C12 was obtained from the Riken Cell Bank (Riken, Ibaraki, Japan) and maintained in Dulbecco's modified Eagle's medium (DMEM) (Sigma-Aldrich, St. Louis, MO, USA) supplemented with 20% fetal bovine serum (FBS) and antibiotics.

**Small interfering RNA transfection**

To evaluate the effect of p21 knockdown, Lipofectamine 2000 (Invitrogen, Carlsbad, CA, USA) was used to transfect C2C12 cells with a p21 siRNA (Invitrogen) or negative control siRNA (Invitrogen) according to the manufacturer’s protocol. In brief, 1 day before transfection, cells were plated on a 12-well plate in growth medium. Then, 50 pmoles of siRNA and Lipofectamine 2000 complexes were prepared and added to each well. After 24 h of transfection, the complexes were removed, and fresh medium containing heat-inactivating 20% FBS as a normal growth medium for myoblast or heat-inactivating 5% horse serum (HS; COSMO BIO Co., Ltd, Tokyo, Japan) as a differentiation medium for myoblast was added. After medium exchange for 3 days, each mRNA was isolated and quantitative RT-PCR was performed for the evaluation of p21 expression. The value for the control siRNA group cultured in 20% FBS was set as one (n = 3). Primer sequences used for Cyclin D1 quantitative RT-PCR were as follows: sense 5′- ATG TCC AAT CCT GGT GAT GTC C -3′; anti-sense 5′- TCA GGG TTT TCT CTT GCA GAA -3′.

**Water-soluble tetrazolium salt assay**

We performed water-soluble tetrazolium salt (WST) assay using the Cell Counting Kit-8 (Dojindo, Kumamoto, Japan), according to the manufacturer’s protocol. In brief, we used 96-well plates and each well was seeded with 5000 cells and filled with 100 µL of medium. Each plate was cultured in CO_2_ incubator at 37°C before WST assay evaluation. For the WST assay, each well was supplemented with 10 µL of WST for 3 hours in CO_2_ incubator at 37°C before spectrophotometric evaluation. Conversion of WST to formazan was spectrophotometrically measured at 450 nm. Total cell viability for each group was expressed as the n-fold difference from the control siRNA group cultured in 20% FBS. The value for the control siRNA group cultured was set at one (n = 3).

**Statistical Analysis**

Statistical analysis was performed using the SPSS software package (IBM, Chicago, Illinois, USA). Comparison of p21 mRNA expression levels and cell viability between control-siRNA and p21-siRNA transfected cells were analyzed by Mann Whitney U-test. All values are presented as mean ± standard error. Results with p < 0.05 were considered statistically significant.

**Supplementary results**

**Differences in p21 mRNA expression in control siRNA or p21 siRNA transfected cells grown in two different media**

In control siRNA transfected cells, p21 mRNA expression was significantly increased in the differentiation medium. The knockdown effect of p21 siRNA was even confirmed in the differentiation medium. These findings indicated that p21 expression increases during muscular differentiation (S1 Fig.).

**p21 knockdown increased cell proliferation**

In control siRNA transfected cells, cell proliferation in differentiation medium was lower than that in growth medium. However, in p21 siRNA transfected cells, cell proliferation significantly increased in both media. These findings indicated that p21 knockdown maintained cell proliferation even in the differentiation medium (S2 Fig.).
